# Increase of a Fibrinolytic Enzyme Production through Promoter Replacement of *aprE3-5* from *Bacillus subtilis* CH3-5

**DOI:** 10.4014/jmb.2103.03027

**Published:** 2021-05-07

**Authors:** Zhuang Yao, Yu Meng, Huong Giang Le, Se Jin Lee, Hye Sung Jeon, Ji Yeon Yoo, Jeong Hwan Kim

**Affiliations:** 1 Division of Applied Life Science (BK21 Four), Graduate School, Gyeongsang National University, Jinju 52828, Republic of Korea; 2 Institute of Agriculture and Life Science, Gyeongsang National University, Jinju 52828, Republic of Korea

**Keywords:** *Bacillus subtilis*, promoter replacement, gene expression, fibrinolytic enzymes

## Abstract

*Bacillus subtilis* CH3-5 isolated from cheonggukjang secretes a 28 kDa protease with a strong fibrinolytic activity. Its gene, *aprE3-5*, was cloned and expressed in a heterologous host (Jeong *et al*., 2007). In this study, the promoter of *aprE3-5* was replaced with other stronger promoters (P_cry3A_, P_10_, P_SG1_, P_srfA_) of *Bacillus* spp. using PCR. The constructed chimeric genes were cloned into pHY300PLK vector, and then introduced into *B. subtilis* WB600. The P10 promoter conferred the highest fibrinolytic activity, *i.e.*, 1.7-fold higher than that conferred by the original promoter. Overproduction of the 28 kDa protease was confirmed using SDS-PAGE and fibrin zymography. RT-qPCR analysis showed that *aprE3-5* expression was 2.0-fold higher with the P10 promoter than with the original promoter. Change of the initiation codon from GTG to ATG further increased the fibrinolytic activity. The highest *aprE3-5* expression was observed when two copies of the P_10_ promoter were placed in tandem upstream of the ATG initiation codon. The construct with P10 promoter and ATG and the construct with two copies of P10 promoter in tandem and ATG exhibited 117% and 148% higher fibrinolytic activity, respectively, than that exhibited by the construct containing P10 promoter and GTG. These results confirmed that significant overproduction of a fibrinolytic enzyme can be achieved by suitable promoter modification, and this approach may have applications in the industrial production of AprE3-5 and related fibrinolytic enzymes.

## Introduction

Fibrinolytic enzymes secreted by some *Bacillus* spp. have been the subject of many researches owing to their application as potential anti-thrombotic agents [1, 2]. Nattokinase is the most well-known enzyme, and commercially sold as a neutraceutical supplement. The overproduction of fibrinolytic enzymes, such as nattokinase, is important for the development of various products that contain them. To achieve this goal, various methods have been tried including screening of novel strains with strong fibrinolytic activities [3-5], optimizing the cultural conditions [6, 7], construction of host strains where fibrinolytic genes were integrated into the chromosome [8, 9], and improvements of fibrinolytic genes through in vitro mutagenesis [10, 11]. One of the most efficient methods for increasing gene expression is the replacement of the original promoter with a known stronger promoter because an increase in the transcription frequency results in an increase in the production of gene products [12].

Previously, we cloned a gene (*aprE3-5*) encoding the major fibrinolytic enzyme of *B. subtilis* CH3-5, which was isolated from cheonggukjang, Korean fermented soybean food. *aprE3-5* encodes a preproenzyme that yields a mature 28 kDa enzyme. *aprE3-5* was expressed in a heterologous host, *B. subtilis* WB600 [13]. In this study, we constructed chimeric *aprE3-5* genes, wherein the original promoter was replaced with one of the four known strong *Bacillus* spp. promoters. Furthermore, we constructed *aprE* genes wherein the initiation codon was changed from GTG to ATG and two copies of the most efficient promoter, *i.e.*, P10, were placed in tandem upstream of the ATG initiation codon. We found that promoter replacement along with other modifications were effective in achieving the overproduction of AprE3-5 and in increasing the fibrinolytic activity of the host cell.

## Materials and Methods

### Construction of *aprE3-5* Genes with Its Promoter Replaced with Other Promoter

Primers were designed to amplify *aprE3-5* with its -35 and -10 promoter sequences were replaced with those from other *Bacillus* promoters (Table 1). PCR reactions were performed using a MJ mini personal thermal cycler (Bio-Rad, USA). pHY3-5 (pHY300PLK containing *aprE3-5*) was used as the template DNA [13]. The reaction mixture (50 μl) consisted of 1 μl of template DNA, 1 μl of each primer (10 μM), 5 μl of dNTPs (0.25 mM), and 0.5 μl of *ExTaq* DNA polymerase (Takara, Japan). Amplification conditions were as follows: 94°C for 5 min, 30 cycles of 94°C for 30 s, 64°C for 30 s, 72°C for 40 s, and a final extension at 72°C for 5 min.

### Introduction of Chimeric *aprE3-5* Genes into *B. subtilis* WB600

Amplified DNA was digested with BamHI and EcoRI, and ligated with pHY300PLK (4.87 kb, Ap^R^, Tc^R^), an *E. coli*-*Bacillus* shuttle vector. The ligation mixture was used to transform *B. subtilis* WB600 competent cells [18]. Preparation of *B. subtilis* WB600 competent cells and electroporation (200 Ω, 21 kV/cm) were done as reported previously [13]. Transformants (TFs) on LB agar plates with tetracycline (15 µg/ml) were screened for the recombinant plasmids. Plasmid DNA was prepared by using commercial kit (iNtRON Biotechnology, Korea), and DNA sequencing was done. Restriction enzyme digestion and agarose gel electrophoresis were performed according to the standard methods [19].

### Growth and Fibrinolytic Activities of *B. subtilis* TFs

*B. subtilis* TFs were cultivated in LB broth with tetracycline (15 µg/ml) at 37°C with shaking. Aliquots were taken at 12 h intervals, and the OD_600_ values were measured. Culture was centrifuged at 4,000 ×*g* for 10 min at 4°C and the supernatant was used as a crude sample for fibrinolytic activity measurement. Fibrinolytic activity was measured by the fibrin plate method as described previously [20].

### SDS-PAGE and Fibrin Zymography

Supernatants obtained as above were analyzed by SDS-PAGE and fibrin zymography. For SDS-PAGE, proteins (10 µg) in the supernatant was concentrated by TCA precipitation, and loaded onto a 10% acrylamide gel after boiled for 10 min in 4 X SDS sample buffer. For fibrin zymography, supernatant (1 µg) was loaded without TCA concentration. Fibrin gel preparation and fibrin zymography were done as described previously [20]. The Dokdo-marker (EBM-1034, Elpis-Biotech., Korea) was used as the size marker.

### Reverse Transcription (RT)-qPCR Analysis

RNA was prepared from 48 h culture of *B. subtilis* WB600 TF by using Trizol/bead method [21], and treated with RQ1 RNase-free DNase (Promega, USA). RT-PCR was done using one-step RT-PCR premix kit (iNtRON Biotechnology, Korea). *aprE3-5* was amplified by using primer pair in Table 2. The 20 μl reaction mixture consisted of 8 μl of premix, each 1 μl of forward and reverse primer, 1 μl of RNA (200 ng), and 9 μl of DEPC-treated water. The reaction was started by 30 min incubation at 45°C, followed by initial denaturation at 94°C for 5 min. PCR cycles consisted of denaturation at 94°C for 1 min, annealing at 53°C for 1 min, and extension at 72°C for 1 min. A total of 25 cycles were repeated, and the final extension was done at 72°C for 5 min. 16S rRNA gene was used as a control, and primer pair 27F and 1492R were used for the amplification. PCR results were checked by agarose gel electrophoresis using a 1% gel and iVDye 1kb DNA Ladder (GenDepot, USA) as a size marker.

Quantitative real-time PCR was done using the reverse transcription PCR products as the templates. qPCR reactions were performed by using primer pairs in Table 2, and the reaction mixture consisted of 10 μl of SYBR-Green mix (Bio-Rad, USA), 1 μl of each primer, 7 μl of distilled water, and 1 μl of 200-fold diluted cDNA product. The reactions were carried out using an instrument (CFX96, Bio-Rad). The relative gene expression was calculated by quantification cycle (Cq) value with the 2^−ΔΔCT^ method [22]. The 16S rRNA gene was used as a control, and all reactions were repeated 3 times.

### Construction of an *aprE3-5* with 2 Copies of P10 Promoter in Tandem

The primer pairs in Table 3 were used to construct an *aprE3-5* containing 2 copies of P10 promoter in tandem. An *aprE3-5* without its promoter was amplified from plasmid pHY3-5 by PCR using *aprE3-5*-np-F and *aprE3-5*-R primers, and the start codon was replaced from 'GTG' to 'ATG'. The reaction mixture (50 μl) consisted of 1 μl of template DNA, 1 μl of each primer (10 μM), 5 μl of dNTPs (0.25 mM), and 0.5 μl of *ExTaq* DNA polymerase (Takara, Tokyo, Japan). Amplification conditions were as follows: 94°C for 5 min, 30 cycles of 94°C for 30 s, 64°C for 30 s, 72°C for 40 s, and a final extension at 72°C for 5 min. The purified DNA and pHY300PLK were digested with BamHI and EcoRI and ligated by T4 DNA ligase. The ligation mixture was used to transform *E. coli* DH5α competent cells, and the recombinant plasmid, pHYnpE2, without a promoter was obtained.

Following the Megawhop protocol [23], the P10 promoter was cloned into the upstream of *aprE3-5* in pHYnpE2 using MegaP10-F and MegaP10-R primers. The PCR product was digested with *DpnI*, and the resulting plasmid was introduced into *E. coli* DH5α to obtain the plasmid pHYP10E2. Then, another P10 promoter was cloned into the pHYP10E2 using Mega2P10-F and Mega2P10-R primers. The PCR product was digested with *DpnI*, and the resulting plasmid was introduced into *E. coli* DH5α to obtain the plasmid pHY2P10E2. pHYP10E2 and pHY2P10E2 were introduced into *B. subtilis* WB600 competent cells, respectively. The fibrinolytic activity assay, SDS-PAGE, and fibrin zymography were done to check the effect of tandem P10 promoters on the expression of *aprE3-5*.

## Results and Discussion

### Construction of *aprE3-5* Genes with Its Promoter Replaced with Other Promoter

To increase the expression level of *aprE3-5* in a heterologous *Bacillus* host, the -35 and -10 promoter sequences of *aprE3-5* (P_aprE3-5_) were replaced with other strong *Bacillus* promoters without changes in the intervening sequences (Table 1). P_cry3Aa_ is a promoter modified from *cry* promoter of *B. thuringiensis* where the promoter is responsible for the overproduction of crystal proteins (Cry) [24]. The original -35 and -10 sequences of *cry* promoter were replaced with the consensus sequences of σ^A^- dependent promoter of *B. subtilis*, generating P_cry3Aa_ [14]. P_10_ promoter was derived from quorum sensing related promoter P_srfA_ where the -35 sequence (GTGATA) was changed into the conserved sequence (TTGACA) [15]. P_SG1_ (same with P_SG-TTGACA_ in the ref. 16) was derived from P_SG35.1_ where the -35 sequence (TACTAA) was replaced with the consensus sequence (TTGACA) [16]. P_srfA_, has the same -35 (GTGATA) and -10 sequences (TAAACT) of promoter of *srfA* [17]. These promoters were chosen because they do not require any specific inducer, which is expensive for large-scale cultivation and inconvenient, too.

Chimeric *aprE3-5* genes with the replaced -35 and -10 sequences were amplified by PCR (data not shown), and ligated with pHY300PLK. *B. subtilis* WB600 TFs harboring recombinant plasmids were obtained. DNA sequencing confirmed that the replaced -35 and -10 promoter sequences were connected to the 1,146 bp *aprE3-5* structural gene as expected (data not shown).

### Growth and Fibrinolytic Activities of *B. subtilis* WB600 TFs

**Fig. 1:**
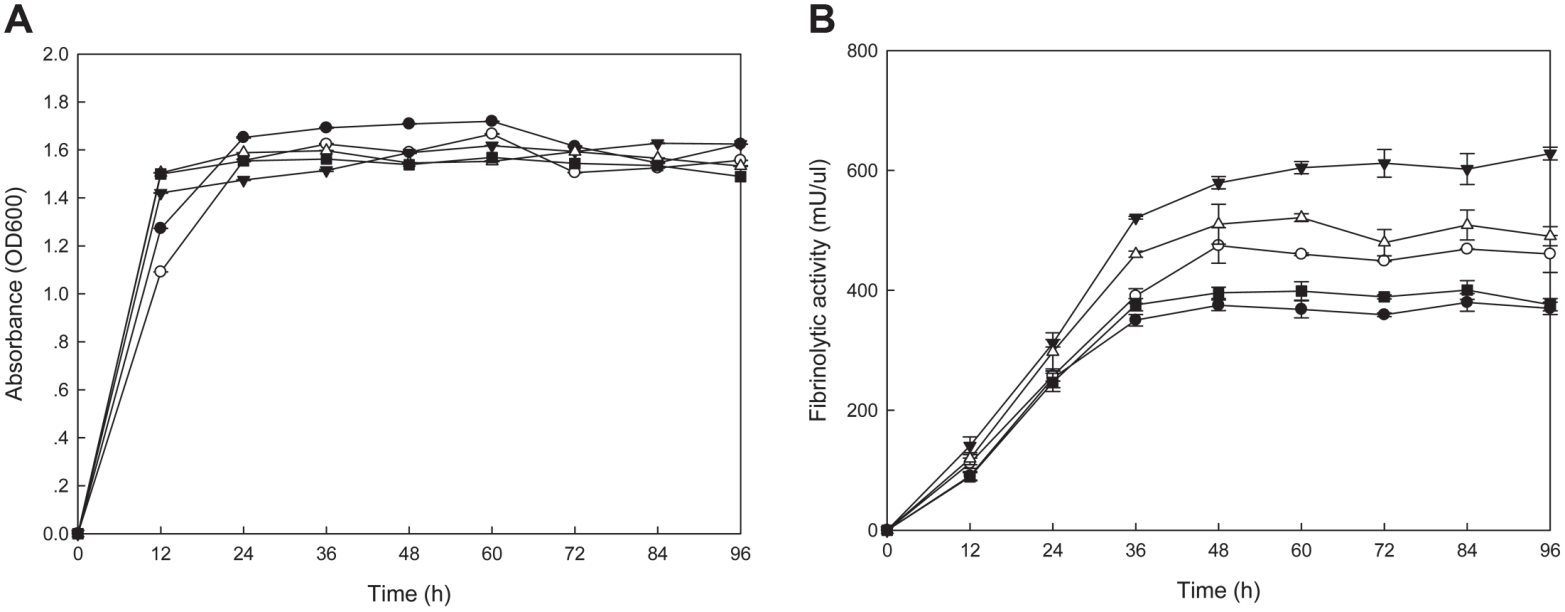
Growth (A) and fibrinolytic activities (B) of *B. subtilis* WB600 TFs. *B. subtilis* TFs were cultivated for 96 h at 37°C in LB broth and the growth (OD_600_) and fibrinolytic activities were measured at 12 h intervals. -●-, *B. subtilis* WB600 [pHY3-5]; -○-, *B. subtilis* WB600 [pHYsrfA]; -▼-, *B. subtilis* WB600 [pHYP10]; -△-, *B. subtilis* WB600 [pHYPSG]; -■-, *B. subtilis* WB600 [pHYPcry3A].

*B. subtilis* WB600 TFs harboring different plasmid constructs (original *aprE3-5* gene and 4 chimeric genes) were inoculated into LB broth and cultured with shaking at 37°C for 96 h. All strains grew well and OD_600_ values reached 1.5-1.7 after 24 h incubation, and the growth curve of each strain was similar (Fig. 1A). Culture carrying P_aprE3-5_ showed fibrinolytic activity (FA) of 369.96 U/ml at 96 h of incubation whereas those of culture carrying P_cry3A_, P_srfA_, P_10_, or P_SG1_ were 376.22, 460.85, 628.15, or 490.23 U/ml, respectively (Fig. 1B). Except the strain carrying P_cry3Aa_, other strains showed significantly higher activities than the strain carrying the original promoter. The strain carrying P10 promoter showed the highest activity (628.15 U/ml), and the activity was 1.7 fold higher than that of the original strain (369.96 U/ml) at 96 h time point. The strain carrying P_SG1_ showed 1.3 fold higher activity. All strains showed similar pattern in fibrinolytic activity changes during the 96 h of incubation. The activities increased rapidly during the first 36 h, and then increased gradually. The highest activities observed between 48 and 60 h. However, the activity increased continuously until 96 h in the strain carrying pHYP10 (pHY300PLK with P10 promoter).

### SDS-PAGE and Fibrin Zymography

**Fig. 2:**
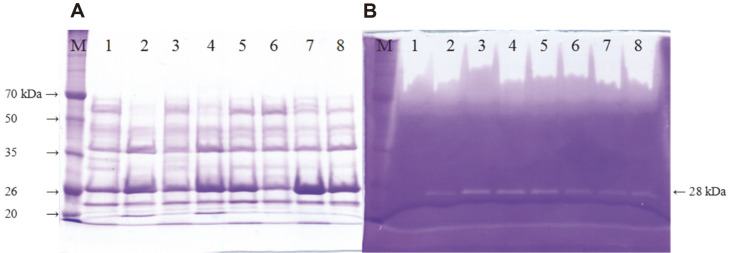
Coomassie blue stained gel (A) and fibrin zymogram (B) of culture supernatant from *B. subtilis* WB600 TFs. M, Dokdo-marker (EBM-1034); lane 1, *B. subtilis* WB600 [pHY3-5] at 48 h; 2, at 96 h; 3, *B. subtilis* WB600 [pHYPsrfA] at 48 h; 4, at 96 h; 5, *B. subtilis* WB600 [pHYPSG] at 48 h; 6, at 96 h; 7, *B. subtilis* WB600 [pHYP10] at 48 h; 8, at 96 h.

Supernatant samples prepared at 48 h and 96 h were analyzed by SDS-PAGE and fibrin zymography using 10%acrylamide gels (Fig. 2). Four bands of 24, 28, 38 and 60 kDa in size were observed on a coomassie blue stained gel (Fig. 2A) and one band (28 kDa) was detected on a fibrin gel (Fig. 2B). The 28 kDa protein was the mature form of AprE3-5. Culture carrying P_10_ promoter showed the strongest band intensity for 28 kDa protein (Fig. 2A, lanes 7, 8). The results indicated that AprE3-5 was overproduced from P_10_ promoter compared to other promoters. Similarly, the top regions of lanes 7 and 8 showed larger transparent areas than others. The big transparent region was suspected to be caused by binding of fibrinolytic enzymes to fibrin in the gel [25], and the size reflects the amount of the fibrinolytic enzymes in the sample. These results were consistent with the fibrinolytic activities of cultures (Fig. 1B).

### Reverse Transcription-qPCR Analysis

**Fig. 3:**
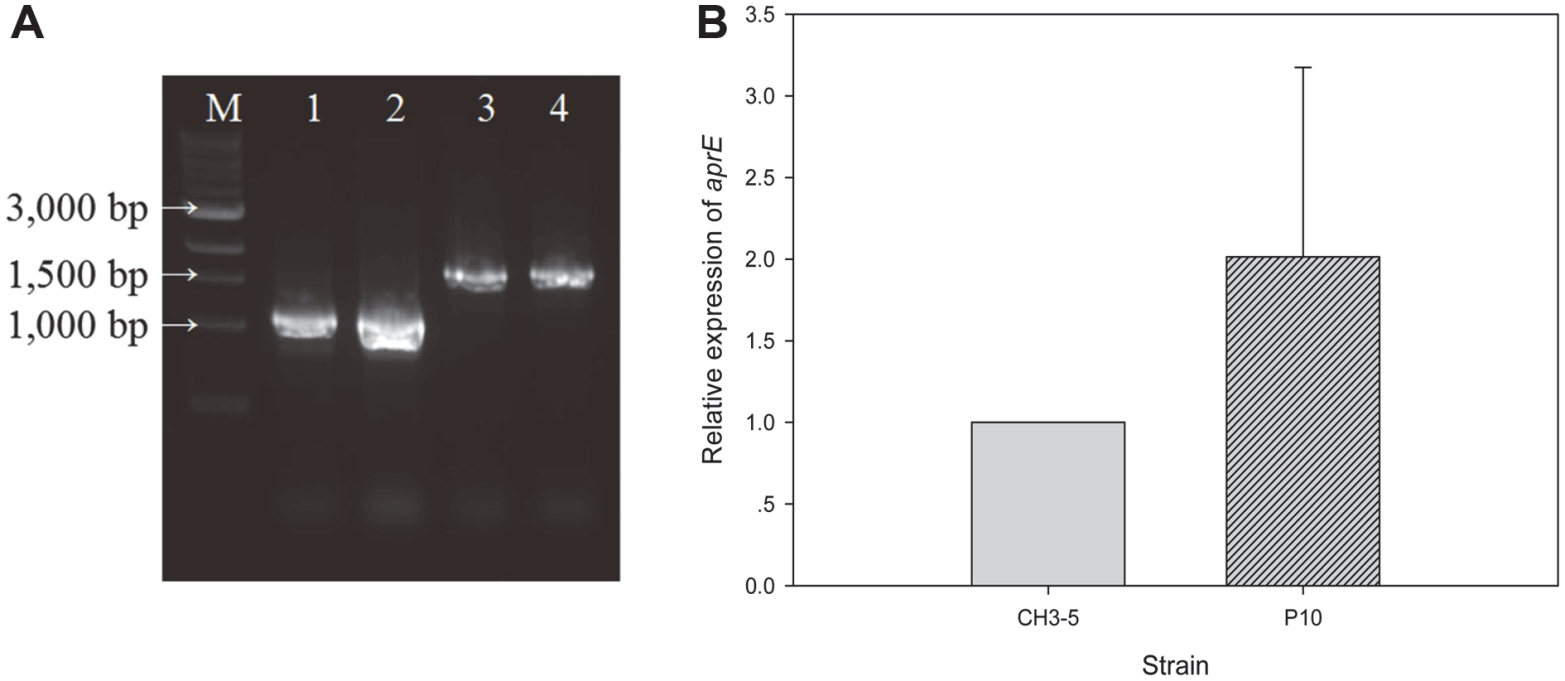
Reverse Transcription (RT)-PCR (A) and the relative expression levels of *aprE3-5* by its own promoter and replaced P10 promoter (B). M, iVDye 1kb DNA Ladder; 1-2, *aprE3-5*, RT-PCR product of *B. subtilis* WB600 [pHY3- 5] (lane 1), and *B. subtilis* WB600 [pHYP10] (lane 2); 3-4, 16S rRNA gene, RT-PCR product of *B. subtilis* WB600 [pHY3-5] (lane 3), and *B. subtilis* WB600 [pHYP10] (lane 4).

RT-PCR was performed with RNA samples to confirm the *aprE3-5* mRNA content in different samples. The expected amplified size of *aprE3-5* was 1 kb. The amplified size of 16S rRNA gene was 1.5 kb. Agarose gel electrophoresis results confirmed 2 cDNA fragments with the matching sizes (Fig. 3A). The cDNA fragments in lane 1 and 2 were amplified *aprE3-5*, around 1 kb, and the cDNA fragments in lane 3 and 4 were amplified 16S rRNA gene, around 1.5 kb. The results showed that the concentration of *aprE3-5* mRNA from the P_10_ carrying strain (lane 2) was significantly higher than that from the original strain (lane 1). The 16S rRNA gene concentrations were the same. The results showed qualitatively that the P_10_ promoter increased the frequency of transcription of *aprE3-5*.

Quantitative real-time PCR analysis was performed using the reverse transcription product as template to quantitatively analyze the effect by P_10_ promoter. Using 16S rRNA gene as a control, the relative expression level of *aprE3-5* was calculated using the 2^−ΔΔCT^ method. The expression level of original strain was set to 1. The expression of *aprE3-5* gene by P_10_ promoter was significantly increased. At 48 h of incubation, the expression level was 2.01-fold higher than that by the original promoter (Fig. 3B). The fibrinolytic activity of strain carrying P_aprE3-5_ or P_10_ was 375.15 U/ml and 579.33 U/ml, respectively at 48 h of incubation. The strain carrying P_10_ promoter showed 1.54 fold higher fibrinolytic activity than that from the strain carrying the original promoter. The difference in gene expression matched with the fibrinolytic activities of cultures.

The -35 sequence of P_10_ was TTGACA, identical with the consensus -35 sequence whereas that of the original *aprE3-5* promoter is TCTACT. The -10 sequence of original *aprE3-5* promoter and P_10_ are TACAAT and TAAACT, respectively. The -10 consensus sequence is TATAAT. Therefore the -10 sequence of original *aprE3-5* promoter is more conserved than that of P_10_, and the results indicated that -35 sequence might contribute more to the overall promoter strength. Overproduction of valuable metabolites such as fibrinolytic enzymes can be achieved by many different methods, and the replacement of original promoter with stronger promoter is one option, which can be applied quickly and easily.

### Construction of *aprE3-5* with 2 Copies of P10 Promoter in Tandem

**Fig. 4:**
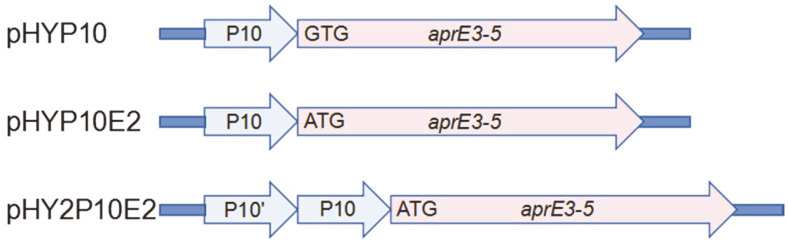
The schematic diagram of the expression cassettes. pHYP10, pHY300PLK containing the *aprE3-5* where the original promoter was replaced with -35 and -10 sequences from P10 promoter. pHYP10E2, pHYP10 where the start codon was changed from GTG to ATG. pHY2P10E2, pHYP10E2 where an additional P10 promoter was placed in tandem.

**Fig. 5:**
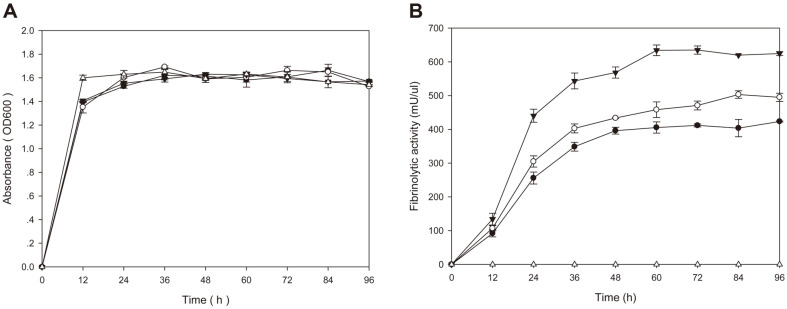
Growth (A) and fibrinolytic activities (B) of *B. subtilis* WB600 TFs. *B. subtilis* TFs were cultivated for 96 h at 37°C in LB broth and the growth (OD_600_) and fibrinolytic activities were measured at 12 h intervals. -●-, *B. subtilis* WB600 [pHYP10]; -○-, *B. subtilis* WB600 [pHYP10E2]; -▼-, *B. subtilis* WB600 [pHY2P10E2]; -△-, *B. subtilis* WB600 [pHY300PLK].

Bacillus strains harboring pHYP10, pHYP10E2, pHY2P10E2, or pHY300PLK (negative control), were obtained (Fig. 4), and cultivated in LB broth. Growth and fibrinolytic activities were measured (Fig. 5). All strains grew well, and showed the same absorbance values (600 nm) at 96 h (Fig. 5A). *B. subtilis* WB600 carrying pHY2P10E2 showed the highest fibrinolytic activity (624.6 mU/μl) at 96 h (Fig. 5B). Cells carrying pHYP10E2 was the next, 495.0 mU/μl. Cells carrying pHY10 showed the activity of 423.3 mU/ml. The activity of the strain carrying pHYP10E2 (ATG start codon) was 117% higher than that of the strain carrying pHYP10, indicating that ATG was better than GTG for gene expression. The activity of the strain carrying pHY2P10E2 was 148% higher than that of the strain carrying pHYP10. The results indicated that 2 copies of P10 promoter in tandem further improved the gene expression level of *aprE3-5* in *B. subtilis*.

**Fig. 6:**
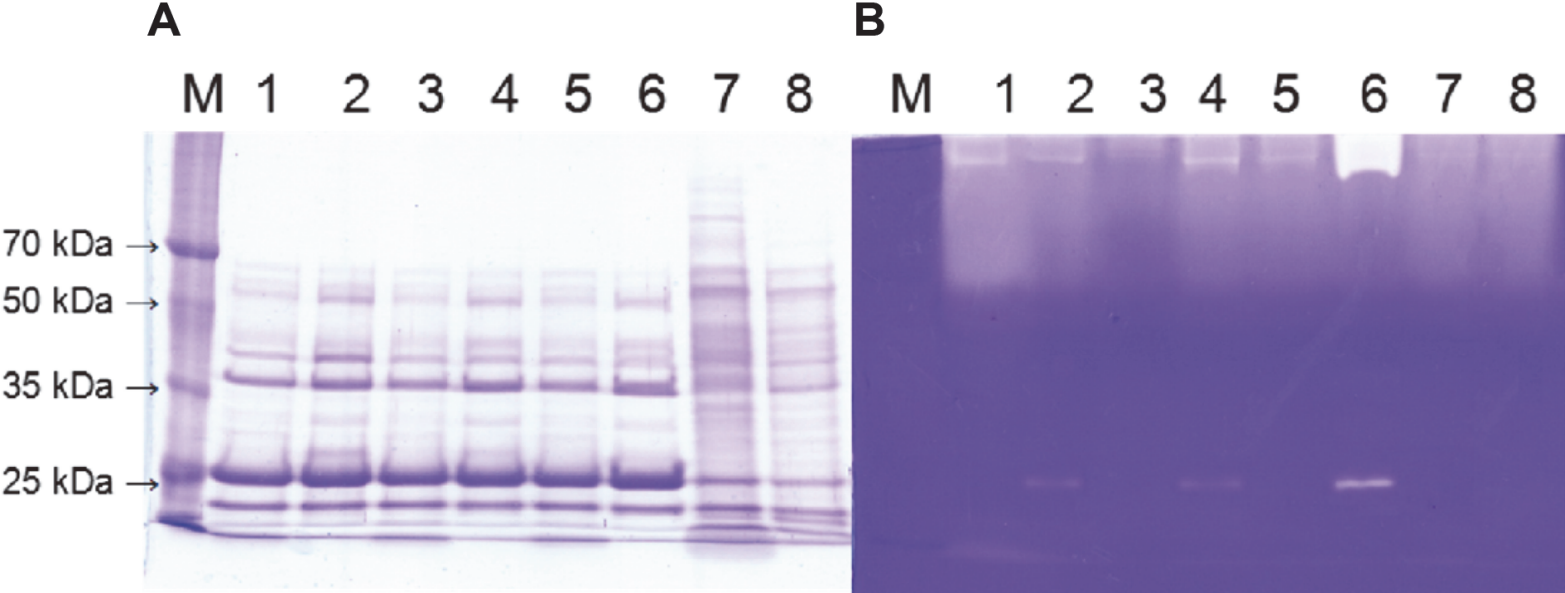
Coomassie blue stained gel (A) and fibrin zymogram (B) of culture supernatant from *B. subtilis* WB600 TFs. M, Dokdo-marker (EBM-1034); lane 1, *B. subtilis* WB600 [pHYP10] at 12 h; 2, at 96 h; 3, *B. subtilis* WB600 [pHYP10E2] at 12 h; 4, at 96 h; 5, *B. subtilis* WB600 [pHY2P10E2] at 12 h; 6, at 96 h; 7, *B. subtilis* WB600 [pHY300PLK] at 12 h; 8, at 96 h.

SDS-PAGE and fibrin zymography were done for culture supernatants obtained at 12 h and 96 h. Four bands of 24, 28, 38, and 60 kDa were observed on the gel stained with coomassie brilliant blue (Fig. 6A). The 28 kDa band was the most obvious, indicating that a large amount of AprE3-5 was produced. On the fibrin zymogram (Fig. 6B), the size of transparent zone at the top of a fibrin gel reflected the fibrinolytic activity of a sample. The sizes of the transparent areas at the top of lanes 1, 3, and 5 were similar with each other, indicating that the activity difference between samples at 12 h was not significant. But the bands with a size of 28 kDa were observed at 96 h samples (lane 2, 4, and 6), indicating that AprE3-5 production occurred at late growth phase or early stationary phase. Especially the clear zone of lane 6 was the largest, indicating that pHY2P10E2 conferred the highest fibrinolytic activity to *B. subtilis* host. More directly, the band intensity of the 28 kDa protein was the strongest in lane 6, 96 h sample from *B. subtilis* carrying pHY2P10E2. The results were consistent with the fibrinolytic activity measurements of the cultures (Fig. 5B).

We successfully showed that the tandem P10 promoter increased the production of AprE3-5. In order to increase the production of AprE3-5 even further, it is necessary to conduct more researches on other elements which also might be important for overproduction of AprE3-5. These include the optimization of Shine-Dalgarno sequence, adjustments of the length of intervening sequence between -35 and -10 promoter sequences, and the use of transcription terminator [26]. Further studies are necessary on these topics in addition to optimization in media composition and cultural conditions.

## Figures and Tables

**Table 1:** Primers used in this study.

Primers	Sequences	References
	Restriction site -35 -10	
aprE3-5-F	5’-CGCGGATCCGGG**TCTACT**AAAATATTATTCCATCTAT**TACAAT**AAATTC -3’	13
Pcry3A-F	5’-CGCGGATCCGGG**TTGCAA**AAAATATTATTCCATCTAT**TAAGCT**AAATTC -3’	14
P10-F	5’-CGCGGATCCGGG**TTGACA**AAAATATTATTCCATCTAT**TAAACT**AAATTC -3’	15
PSG1-F	5’-CGCGGATCCGGG**TTGACA**AAAATATTATTCCATCTAT**TACAAT**AAATTC -3’	16
PsrfA-F	5’-CGCGGATCCGGG**GTGATA**AAAATATTATTCCATCTAT**TAAACT**AAATTC -3’	17
*aprE3-5*-R	5'- GCGAATTCGAGAACAGAGAAGCCGCT -3'	13

The restriction site was underlined: *BamHI* (forward primer) and *EcoRI* (reverse primer).The -35 and -10 promoter regions were in bold and underlined.

**Table 2:** Primers used for reverse transcription PCR.

PCR reaction	Genes name	Primer pairs	Sequences	Expected size
RT-PCR	*aprE3-5*	aprE-RT-F aprE-RT-R	5'-TGGATCAGCTTGTTGTTTGCG-3' 5'-GGGTGCTTAGAAAGGATTAGC-3'	1 kb
	16S rRNA	27F 1492R	5'-AGAGTTTGATCCTGGCTCAG-3' 5'-GGTTACCTTGTTACGACTT-3'	1.5 kb
qRT-PCR	*aprE3-5*	aprE-qRT-F aprE-qRT-R	5'-AACAGCAGCAACCAAAGAGC-3' 5'-TCGGGTGCTTAGAAAGGAT-3'	178 bp
	16S rRNA	16S-qRT-F 16S-qRT-R	5'-GAGTGACAGCTGGTGCATGGT-3' 5'-TTGTCACCGGCAGTCACCTTA-3'	160 bp

**Table 3:** Primers used for construction of an *aprE* with tandem P10 promoter.

Primers	Sequences	References
aprE3-5-np-F	5’-CTGGATCCTCTTAAAAGGAGAGGGTAAAGAATGAGAAGCA-3’’	This study
aprE3-5-R	5'- GCGAATTCGAGAACAGAGAAGCCGCT -3'	13
MagaP10-F	5’-AAGCTTCTAGAGATCTGCAGGTCGACGGG**TTGACA**AAAATATTATTCCATCTAT**TAAACT**AAATTCACAGAATAGTCTTT -3’	This study
MagaP10-R	5’-TTTTAAGAGGATCCAGAGTAGACTTACTTAAAAGACTATTCTGTGAATTTAGTTTAATAGATGGAATAATATTTTTGTC -3’	This study
Maga2P10-F	5’-GGCGGAGCCTATGGAAAAACGCTTTGCCC**TTGACA**AAAATATTATTCCATCTAT**TAAACT**AAGCTT -3’	This study
Maga2P10-R	5’-CCCGTCGACCTGCAGATCTCTAGAAGCTTAGTTTAATAGATGGAATAATATTTTTGTCAAGGGCAA-3’	This study

The restriction site was underlined: BamHI (forward primer) and EcoRI (reverse primer).The -35 and -10 promoter regions were in bold and underlined.
